# Complete Genome Sequence of *Cronobacter sakazakii* Strain CMCC 45402

**DOI:** 10.1128/genomeA.01139-13

**Published:** 2014-01-16

**Authors:** Zhijing Zhao, Lei Wang, Bin Wang, Haoyu Liang, Qiang Ye, Ming Zeng

**Affiliations:** Division of Enteric Bacterial Vaccines, National Institutes for Food and Drug Control, Beijing, Chinaa; TEDA School of Biological Sciences and Biotechnology, Nankai University, Tianjin, Chinab; Division of Respiratory Bacterial Vaccines, National Institutes for Food and Drug Control, Beijing, Chinac

## Abstract

*Cronobacter sakazakii* is considered to be an important pathogen involved in life-threatening neonatal infections. Here, we report the annotated complete genome sequence of *C. sakazakii* strain CMCC 45402, obtained from a milk sample in China. The major findings from the genomic analysis provide a better understanding of the isolates from China.

## GENOME ANNOUNCEMENT

*Cronobacter* spp. (formerly *Enterobacter sakazakii*) are Gram-negative opportunistic pathogens that cause life-threatening infections, such as meningitis, sepsis, bacteremia, and necrotizing enterocolitis in neonates, children, and immune compromised adults, particularly older persons (1, 2). Cases of meningitis and necrotizing enterocolitis following *Cronobacter sakazakii* infection both have high mortality rates of 40 to 80% and 10 to 55%, respectively (3, 4). *Cronobacter* spp. have been recovered from a wide variety of foods, including powdered infant formula (PIF), weaning foods, meat, cheese, vegetables, grains, herbs, spices, milk, teas, dried infant and adult cereals, tofu, chocolate, and pastas, from environments, such as factories and households, and even from insects (5–7). Several studies have shown the prevalence of *C. sakazakii* in dairy products in China to be from 1.22 to ~66.67% (8). These organisms are known for their stress response. Until the present, little has been known about the mechanisms of pathogenicity in *Cronobacter* species. In the present study, *C. sakazakii* CMCC 45402 was isolated from milk samples in China. To enhance our understanding of this microorganism, whole-genome and plasmid sequencing were performed.

Whole-genome sequencing of *C. sakazakii* CMCC 45402, which was isolated from the milk samples in China, was performed with a combined strategy using a 454 sequencing and Solexa paired-end sequencing technology. Genomic libraries containing 8-kb inserts were constructed, and 269,508 paired-end reads and 94,334 single-end reads were generated using the 454 GS FLX+ system, giving 24.7-fold coverage of the genome. Automatic assembly was done using the Newbler 2.6 software (454 Life Sciences, Branford, CT) and yielded 6 large scaffolds, including 46 nonredundant contigs. A total of 8,292,276 paired-end reads (500-bp library) were generated to reach a depth of 359-fold coverage with the Illumina genome analyzer IIx (Illumina, San Diego, CA), and they were mapped to the scaffolds using the Burrows-Wheeler Aligner (BWA). Sequence gaps were ﬁlled through the sequencing of PCR products generated from an ABI 3730. Prediction and annotation of protein-encoding genes were performed as described previously.

The complete genome sequence of *C. sakazakii* CMCC 45402 contains a circular 4,377,544-bp chromosome with a G+C content of 56.89% and two circular plasmids of 126,488 bp and 55,913 bp.

In total, there are 4,249 predicted genes in the chromosome, including 4,160 protein-encoding genes, 82 tRNA-encoding genes, and 7 rRNA-encoding genes. Of the 4,160 predicted protein-encoding genes, biological roles were assigned to 2,658 genes (63.89%) with similarity to proteins of known function according to the COG scheme. Of the total, 1,747 (42%) predicted coding sequences matched gene products of unknown function from other species, and 156 (3.6%) had no database match.

Five hundred sixty (13.5%) genes of the predicted protein-encoding genes are involved in metabolic pathways, and 253 (6.1%) participate in the biosynthesis of secondary metabolites. These predicted genes also participate in pathways, such as microbial metabolism in diverse environments, purine metabolism, and the ABC transporter pathway.

### Nucleotide sequence accession numbers.

The sequence and annotation of the *C. sakazakii* CMCC 45402 genome have been deposited in GenBank under the accession no. CP006731, accompanied by two plasmid sequences (accession no. CP006732 and CP006733).
